# A Focus on the Death Kinetics in Predictive Microbiology: Benefits and Limits of the Most Important Models and Some Tools Dealing with Their Application in Foods

**DOI:** 10.3390/foods4040565

**Published:** 2015-10-12

**Authors:** Antonio Bevilacqua, Barbara Speranza, Milena Sinigaglia, Maria Rosaria Corbo

**Affiliations:** Department of the Science of Agriculture, Food and Environment, University of Foggia, Via Napoli 25, 71122 Foggia, Italy; E-Mails: barbara.speranza@unifg.it (B.S.); milena.sinigaglia@unifg.it (M.S.); mariarosaria.corbo@unifg.it (M.R.C.)

**Keywords:** inactivation kinetics, use, drawbacks, software

## Abstract

Predictive Microbiology (PM) deals with the mathematical modeling of microorganisms in foods for different applications (challenge test, evaluation of microbiological shelf life, prediction of the microbiological hazards connected with foods, *etc*.). An interesting and important part of PM focuses on the use of primary functions to fit data of death kinetics of spoilage, pathogenic, and useful microorganisms following thermal or non-conventional treatments and can also be used to model survivors throughout storage. The main topic of this review is a focus on the most important death models (negative Gompertz, log-linear, shoulder/tail, Weibull, Weibull+tail, re-parameterized Weibull, biphasic approach, *etc.*) to pinpoint the benefits and the limits of each model; in addition, the last section addresses the most important tools for the use of death kinetics and predictive microbiology in a user-friendly way.

## 1. Introduction: A Focus on Predictive Microbiology

The origin of Predictive Microbiology (PM) is linked to research by Bigelow [1], Bigelow and Esty [2] and Esty and Meyer [3]; they proposed the famous log-linear equation to model heat inactivation of *Clostridium botulinum* [4]. Although many papers focused on mathematical models for pathogen inactivation, the term PM was introduced by Robert and Jarvis [5].

Currently, PM is usually referred as a tool/theory applied to improve food safety and quality and there are many models and statistical tools dealing with pathogens, toxins, spoilage microorganisms, thermal, and non-thermal processes. Traditionally, models of predictive microbiology are split up into three groups: (1) primary models, which represent population density (or number) *versus* time; (2) secondary models, describing the effects of some environmental and technological factors on the parameters of primary models (lag phase, growth and death rate, population density in the stationary phase); (3) tertiary models, which are computer tools able to give an output on microbial growth/death as a function of some parameters defined by the end-user.

This paper focuses on the primary models, namely as applied to inactivation trends, focusing on the most important inactivation models and providing some examples of user-friendly statistical tools that can fit inactivation data.

## 2. Overview of Death Equations

### 2.1. Log-Linear Model

A log-linear equation is the most simple approach to describe inactivation kinetics; it is based on the assumption that there is a negative and linear correlation between cell count and lethal treatment/inactivation rate as follows:

(1) $$N=N_{0}-kt$$

where *N* and *N*_0_ are respectively cell count over time and the initial cell count; *t* is the independent variable (time, temperature, pressure, *etc.*) and *k* the inactivation rate. The classical equation of exponential inactivation was proposed by Esty and Meyer [3] and Esty and Williams [6], reading as follows:

(2) $$N=N_{0}e^{-kt}$$

This general equation was cast in the log-linear form as follows:

(3) $$\log N=\log N_{0}-\frac{k_{\max}t}{\ln10}$$

Ball and Olson [7] introduced the idea of the decimal reduction value or first reduction time and used the symbol *D*; thus, the equation was proposed in the most widely known form:

(4) $$\log N=\log N_{0}-\frac{t}{D}$$

where *N* and *N*_0_ are the population at the time *t* and the initial cell number, respectively; *k*_max_ the inactivation rate; *t*, the time and *D* the decimal reduction value. This equation remains the most used approach for the evaluation of the thermal sensitivity of bacteria and fungi, though it operates on the main assumption that all cells in a population possess the same thermal resistance [8,9], thus it does not take into account variability at the cell level.

In its classical form, the independent variable is time (duration of thermal treatment); however, it can be also used to model the effect of pressure or chemicals on some microorganisms.

This equation works very well in describing the inactivation kinetics of simple systems (e.g., laboratory media) or for single strain populations. It fails in fitting data from complex environments and for populations showing a significant deviation from linearity. This phenomenon occurs when the lethal treatment needs to achieve a critical breakpoint in order to exert a significant effect on the target organisms.

### 2.2. Shoulder-Tail and Negative Gompertz Equations

Different authors have reported that inactivation kinetics could be described by a negative sigmoid or by a non-linear equation covering at least three different steps or phases: a shoulder (no decrease in cell count), an exponential death phase (described by the inactivation rate *k*_max_) and a tail, *i.e.*, a residual population [10]. Before describing the functions to fit this trend, it is prudent to define the biological meaning of shoulder and tail.

Geeraerd *et al.* [10] reported at least four different reasons for the presence of a shoulder in a death kinetic:

i) Microorganisms are organized in clumps. The shoulder is the time before all but one organism in such a clump has been killed.
ii) Cells tend to counterbalance the effect of a lethal treatment, thus the shoulder is the period when cells are able to resynthesize a critical component and death occurs only when the rate of destruction exceeds the rate of synthesis.
iii) A shoulder could describe the protective effect of the medium or some components (fats, proteins) on cells.
iv) A shoulder may describe a kind of cumulative injury that must occur before cell inactivation.

On the other hand, a tail describes what happens in the final stage of inactivation kinetics [11]; it implies the existence of a sub-resistant population. This fraction of population can be described according to a vitalistic approach or by using one of two different mechanistic theories [12], *i.e.*,:

i) Vitalistic approach: this sub-population is very resistant to heat/inactivation treatment.
ii) Mechanistic approach. Theory I: the tail is a “normal” trait of an inactivation kinetic, as it describes a sub-population inaccessible to or adapted to the lethal treatment.
iii) Mechanistic approach. Theory II: the tail is an artifact, because the residual sub-population is genetically more resistant or does not receive the same lethal dose.

The shoulder/tail model by Geeraerd *et al.* [10] comprises shoulder and tail steps; it is completely described by two parameters (*k*_max_ and *N_res_*, respectively inactivation rate and residual sub-population) and two states (*N* and *C_c_*):

(5) $$\frac{dN}{dt}=-kN$$

(6) $$\frac{dC}{de}=-k_{\max}C_{c}$$

(7) $$k=k_{\max}\left(\frac{1}{1+C_{c}}\right)\left(1-\frac{N_{res}}{N}\right)$$

This equation has four degrees of freedom: two parameters (*k*_max_ and *N_res_*) and two initial states (*N*(0) and *C_c_*(0)); it encompasses log-linear inactivation by the selection of a very low value for *C_c_*(0) and *N_res_*, thus implying the lack of a shoulder and tail.

The couple of differential equations has an explicit solution [10,12] and reads as follows, after substituting *C_c_*(0) (the initial concentration of the critical component) by $e^{k\max SL}-1$ with SL (time units), a parameter representing the shoulder length:

(8) $$N\left(t\right)=\left(N_{0}-N_{res}\right)e^{-k_{\max}t}\left(\frac{e^{k_{\max}SL}}{1+\left(e^{k_{\max}SL}-1\right)e^{-k_{\max}t}}\right)+N_{res}$$

This model offers some benefits: it can be used both for deterministic and mechanistic approaches, it covers many situations (log-linear decay, shoulder/tail shape, shoulder + log decay, log-decay + tail), and does not rely upon the estimation of *N*(0).

Another possibility is the use of the re-parameterized Gompertz equation, cast in its negative form, as suggested by Bhaduri *et al.* [13], Linton *et al.* [14], and Corbo *et al.* [15]. The linear form of the negative Gompertz equation reads as follows:

(9) $$y=k-\Delta \exp\left\{-\exp\left[\left(\frac{d_{\max}e}{\Delta }\right)\left(\alpha -time\right)+1\right]\right\}$$

where *k* and Δ are respectively the initial cell count and the decrease of cell count over time, *d*_max_ is the maximal inactivation rate, and α the shoulder. The benefit of this approach is the possibility of using a single function to model both growth and inactivation; the only change required is in the sign “−” between the parameters *k* and Δ.

Although it has been used worldwide, this approach suffers two drawbacks: (i) in the static version *N*(*t* = 0) is not equal to *N*(0); (ii) the differential equation does explicitly depend on *N*(0) [10].

These limitations could significantly affect data fitting and lead to over- or under-estimation of the parameters.

### 2.3. Weibull

The Weibull model was first cast as a probabilistic-like function, reading as follows:

(10) $$f\left(t\right)=\frac{\beta }{\alpha }{\left(\frac{t}{\alpha }\right)}^{\beta -1}\exp\left(-{\left(\frac{t}{\alpha }\right)}^{\beta }\right)$$

The cumulative distribution of the Weibull function is:

(11) $$F\left(t\right)=\exp\left(-{\left(\frac{t}{\alpha }\right)}^{\beta }\right)$$

Or, for the survival kinetics:

(12) $$\ln S\left(t\right)=-{\left(\frac{t}{\alpha }\right)}^{\beta }$$

where *S* is the ratio *N*/*N*0; β describes the shape of the curve (β = 1, straight line; β < 1, concave curve; β > 1, convex curve). The parameter α modifies the slope but it does not affect the shape [16]. The Weibull equation can be cast in the decimal logarithmic form [17,18]:

(13) logN=logN0−(tδ)p

The parameter δ is the first reduction time and is similar to the *D*-value of the linear thermal inactivation, as it is the time required to attain a 1-log reduction in cell count. *p* is a dimensionless parameter and is referred to as “shape parameter”; it is linked to the geometrical shape of the curve (parameter β of Equation (12)).

Bevilacqua *et al.* [19] slightly modified the classical form of the Weibull equation for the evaluation of the death time (*d.t*.) of a population (*i.e.*, the time after which microorganisms are present at undetectable levels):

(14) logNN0=1−(td.t.)p

Van Boekel [18] and Mafart *et al.* [17] found a strong correlation between δ and p and the dependency of the parameters is probably due to model structure—this link could lead to a systematic bias. Thus, it is possible to use a Weibullian-like function with a fixed-*p* value.

The last modification of the Weibull equation relies upon the decay where a curve trend is followed by a tail; thus, Albert and Mafart [20] described a model able to fit linear, convex, or concave curves followed by a residual sub-population. The model can be written as follows:

(15) $$\log N=\log\left[\left(N_{0}-N_{res}\right)10^{\left(-{\left(\frac{t}{\delta }\right)}^{p}\right)}+N_{res}\right]$$

The four degrees of freedom used are δ (time units), *p* (dimensionless), *N*_0_ and *N_res_*. Convex/concave curves are widespread, thus Weibull can satisfactorily fit thermal or non-thermal decay of many microorganisms.

### 2.4. Biphasic Equation

In the context of thermal inactivation curves as well as for non-thermal approaches [10], inactivation can fit a two-phase decay; *i.e.*, there are two different slopes (*k*1 and *k*2) in the inactivation kinetic.

This trend is generally attributed to the existence of two sub-populations, with different phenotypes. Cerf [21] proposed a model reading as follows:

(16) $$y=y_{0}+\log\left[fe^{-k\max1t}+\left(1-f\right)e^{-k\max2t}\right]$$

where *f* is the fraction of the initial population characterized by the death rate *k*_max1_ and (1 − *f*) the second sub-population (more resistant to lethal treatment), with an inactivation rate *k*_max2_.

An uncommon biphasic trend is one with a preceding biphasic shoulder; Geeraerd *et al.* [9] reparameterized the shoulder/tail equation to better fit this trend. The function is as follows:

(17) $$y=y_{0}+\log\left\{\left[f\exp\left(-k_{\max1}t\right)+\left(1-f\right)\exp\left(-k_{\max2}t\right)\right]\exp\frac{k_{\max1}SL}{1+\left[\exp\left(k_{\max1}SL\right)-1\right]\exp\left(-k_{\max1}t\right)}\right\}$$

### 2.5. Other Models

Some other models can be used to fit data from non-linear trends. This section proposes a short overview on them.

#### 2.5.1. Casolari I

The model of Casolari [11] is based on the mechanistic theory of tailing, which refers to tail as normal trait of thermal inactivation. Another basic idea of the model is that the death of microorganisms is caused by a lethal hit of a water molecule that carries a certain level of energy, higher than a critical break (*E_d_*).

(18) $$N\left(t\right)=N{\left(0\right)}^{\frac{1}{1+B\left(T\right)t}}\;\text{and}\;B\left(T\right)={\left(\frac{N_{A}}{M_{H_{2}O}}\right)}^{2}\exp\left(\frac{-2E_{d}}{RT}\right)$$

with *N*(0) as the initial population, *R* the universal gas constant (8.314 kcal/mL), *T* the absolute temperature (K), *M_H_*_2*O*_ (g/mol) the mass of one mol of water and *N_A_* the Avogadro number (1/mol).

The model describes the tail but not the shoulder.

#### 2.5.2. Casolari II

Casolari [11] proposed a small adaptation of the first equation to model the shoulder. The function reads as follows:

(19) $$N\left(t\right)=N{\left(0\right)}^{\frac{1}{1+B\left(T\right)t^{2}}}\;\text{and}\;B\left(T\right)={\left(\frac{N_{A}}{M_{H_{2}O}}\right)}^{2}\exp\left(\frac{-2E_{d}}{RT}\right)$$

A drawback of the model is the lack of a degree of freedom; moreover, there is a correlation between shoulder and the slope beyond.

#### 2.5.3. Sapru

The model of Sapru [22] was proposed to describe the activation of spores during sterilization; this phenomenon implies a natural increase of activated spores. The function distinguishes a dormant viable population (*N_D_*) and an active population (*N_A_*):

(20) $$\frac{dN_{D}}{dt}=-\left(k_{d1}+K_{a}\right)N_{D}\;\text{with initial condition}\;N_{D}(0)$$

(21) $$\frac{dNA}{dt}=k_{a}N_{D}-k_{d2}N_{A}\;\text{with initial condition}\;N_{A}(0)$$

*where k_d_*_1_ is the first order inactivation constant (1/min) of the dormant population; *K_a_* (1/min) is the first order activation constant of the dormant spores; *k_d_*_2_ (1/min) is the first order inactivation of *N_A_*.

The model can describe tailing, but it suffers some limitations in shoulder estimation.

#### 2.5.4. Whiting

The model of Whiting [23] takes into account the shoulder and describes a death kinetic with two different regions of linearly decreasing populations with the second region showing a less-negative slope.

The general equation of Whiting is as follows:

(22) $$\log\left(\frac{N}{N\left(0\right)}\right)=\log\left(\frac{F_{1}\left(1+\exp\left(-b_{1}t_{1}\right)\right)}{1+\exp\left(b_{1}\left(t-t_{1}\right)\right)}+\frac{\left(1-F_{1}\right)\left(1+\exp\left(-b_{2}t_{1}\right)\right)}{1+\exp\left(b_{2}\left(t-t_{1}\right)\right)}\right)$$

*where b*_1_ is the maximum specific death rate of the major population; *b*_2_ the maximum specific death rate of the subpopulation; *F*_1_ the fraction of initial population in the major population; *t*_1_ is the shoulder.

A major limitation is that *t*_1_ does not visually coincide with a time period where the population is constant.

#### 2.5.5. Xiong

The model assumes a shoulder region (*t_lag_*), followed by a non-linear decrease. The total population is divided over two fractions (*N*_1_ and *N*_2_), differing in heat inactivation constants (*k*_1_ > *k*_2_) [24]. The function is unable to deal with realistic temperature fluctuations [9]. The function is as follows:

$$\left\{ \begin{array}{c} N_{1}\left(t\right)=N_{01}\;\text{with}\;\left(N_{01}\geq 0;t_{0}\leq t\leq t_{lag}\right)\\ \frac{dN_{1}}{dt}=-k_{1}N_{1}\;\text{with}\;\left(t\geq t_{lag}\right) \end{array}\right.$$

(23) $$\left\{ \begin{array}{c} N_{2}\left(t\right)=N_{02}\;\text{with}\;\left(N_{02}\geq 0;t_{0}\leq t\leq t_{lag}\right)\\ \frac{dN_{2}}{dt}=-k_{2}N_{2}\;\text{with}\;\left(t\geq t_{lag}\right) \end{array}\right.$$

with *t*_0_ the initial time, $N\left(t\right)=N_{1}\left(t\right)+N_{2}\left(t\right)$ and $N\left(0\right)=N_{01}+N_{02}$.

Some other inactivation curves can be found in the literature. An extensive description of their benefits and limitations can be found in the paper by Geeraerd *et al.* [9].

Herein we have discussed only the most widely used and user-friendly approaches.

### 2.6. Death Model and Probability

Inactivation models generally describe microbial populations as homogeneous groups; however, Peleg and Cole [25] suggested that this basic assumption is not true. In 1998, they published a paper entitled “Reinterpretation of Microbial Survival Curves” with a special emphasis on heat inactivation. They assumed that a survival curve is the cumulative form of a temporal distribution of lethal events, that is, a cell or spore dies at a specific moment. Thus, it is possible to build a cumulative distribution of death over time and this cumulative distribution is the primary cause affecting the shape of the survival curve and could cause a shift from linear to non-linear death kinetics or from upward to downward curves.

Semi-logarithmic survival curves (log counts *vs*. time) are only reflections of the probabilistic distributions of resistance to heat or to any other kind of stress; these distributions can have different mode, variance, or skewness. Thus, changes in growth or inactivation conditions cause a significant change in the statistical parameters of these functions. This characteristic of semi-logarithmic curves could be of interest and may aid researchers in understanding the underlying mechanism for a particular kind of inactivation.

An overview of the benefits and limitations associated with the approaches discussed herein can be found in Table 1.

**Table 1:** Overview of the benefits and the limitations of the most used models.

	Benefits	Limits
**Shoulder/ tail**	- Possibility to fit data with shoulder and tail - User-friendly - It can be used for the evaluation of shelf life	- It cannot fit data from heterogeneous populations
**Negative Gompertz**	- Possibility to fit data with shoulder and tail - Re-parameterized for the evaluation of the microbiological shelf life and to model a wide range of physicochemical data	- It fails in fitting linear trends - It could lead to over or underestimation of the death rate - It cannot fit data with a tail but without a shoulder and the model gives a negative shoulder phase - It cannot fit data from heterogeneous populations
**Weibull**	- The model can fit a wide range of trends (linear, upward, and downward curves) - There are only two parameters - Re-parameterized for trends with a tail	- It fails in fitting data from non-thermal inactivation or for the decay of some microorganisms under refrigeration with a prolonged shoulder
**Biphasic**	- Fitting of complex trend and inactivation data of heterogeneous populations	- It is not user-friendly

## 3. Tools

As reported by Bassett *et al.* [26], software for PM can be divided into at least three different types: (i) Spreadsheet (generally Excel)-based tools, developed for risk assessment and stochastic simulation; (ii) General simulation software, programming languages, mathematical modeling. These tools require advanced skills; (iii) Tools for Bayesian analysis.

Fitting data from a death kinetic could be referred to as a part of an exposure assessment; modeling can be done through the application of some advanced skills in a variety of software suites, such as SAS, Statistica for Windows, R, Mathematica, Matlab and many others. However, they require the ability to write equations and set the initial conditions for data fitting, as well as a deep knowledge of statistics. Herein, we focus on some Excel add-in components with a user-friendly interface, containing some in-house models. Users should choose the model and/or the initial conditions and then fit the data.

The following sections report on four useful and friendly tools: GInaFit, MLA, AMI, and DMFit.

### 3.1. GInaFit

GInaFit is an add-in Excel component [27]. It was released by Geeraerd *et al.* [9].

The current version covers different models: (1) log-linear curves; (2) log-linear curves with an initial shoulder; (3) log-linear curves with a final tailing; (4) curves with both shoulder and tail; (5) concave and convex curves (Weibullian-like functions); (6) convex/concave curves with a final tailing; (7) biphasic inactivation kinetic with and without a shoulder; (8) curves with a double convex/concave shape.

The significance of the models and parameters is pointed out by the following indices: the Sum of Squared Error, the (Root) Mean Sum of Squared Error, *R*^2^, and adjusted *R*^2^. In addition, the software gives another output, *t*_4D_ (the time needed for a 4 log reduction of the microbial population).

**Figure 1:**
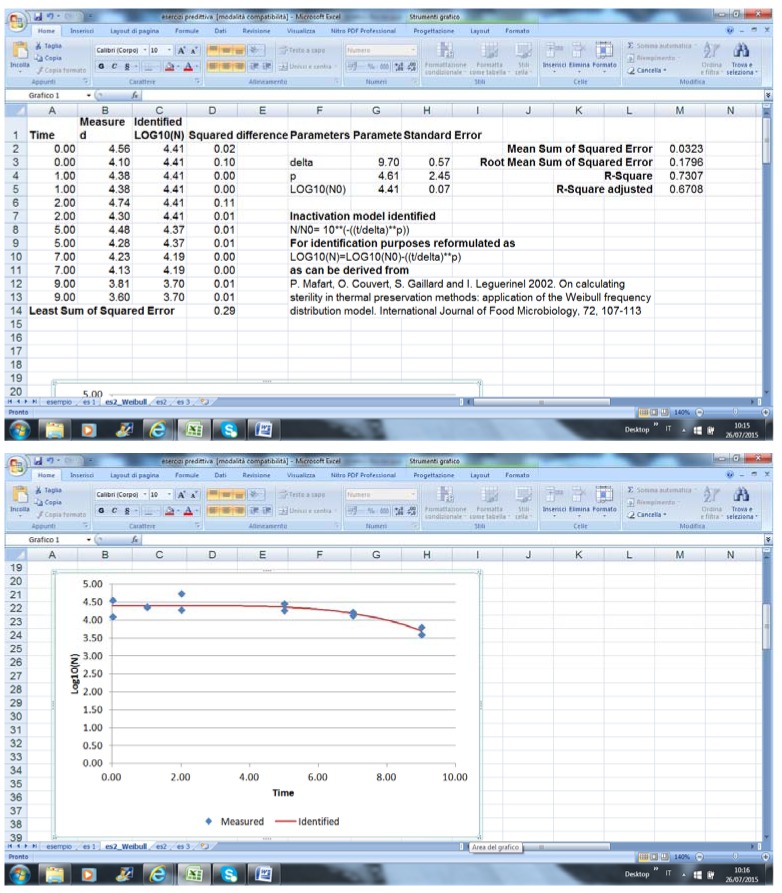
Output of GInaFit.

GInaFit works as an Excel component. After writing or pasting data, the end-user should select the model and run the equation; an example of output is in Figure 1 (data fitting was performed with some arbitrary data).

The output comprises the estimation of predicted values of cell count for each data point, the indices for the significance of the model, the original reference for the model, the equation in the linearized form, and the figure.

**Figure 2:**
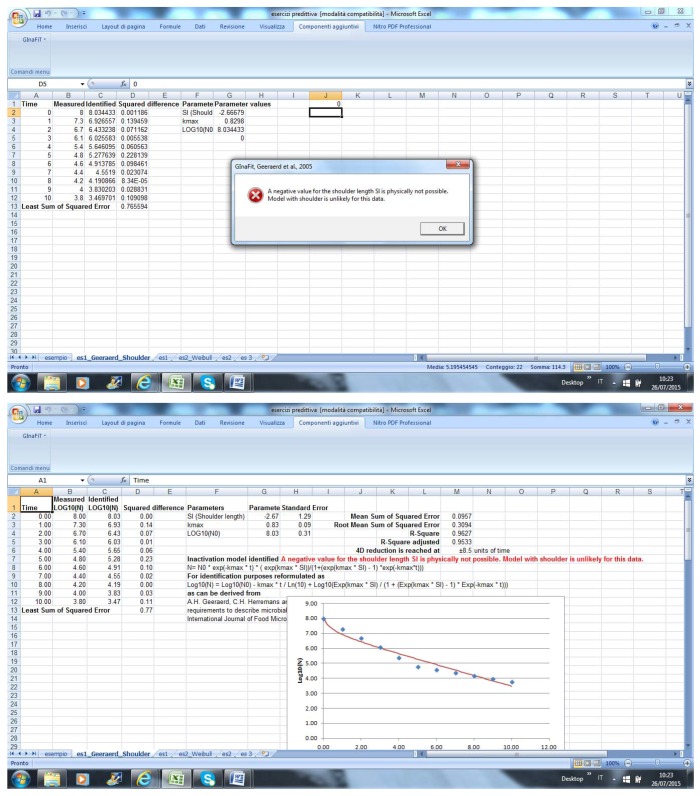
Selection of an incorrect model in GInaFit.

If the end-user selects the wrong model, an error message is returned (Figure 2).

### 3.2. Meat and Livestock Australia (MLA)-Model for Escherichia coli Inactivation in Fermented Meats 

The tool was developed by the Australian Food Safety Centre as a tool for manufacturers to evaluate if the conditions for in-house meat fermentation are sufficient to inactivate *Escherichia coli* [28]. The tool is an Excel file and offers two options: a quick calculator and an advanced tool. The latter is useful if the end-user can import the data from a data logger. The tool is the result of extensive research by the Australian Food Safety Centre. A report on the Institute’s website describes the theoretical background behind the model and the experiments and modeling that form the basis of the software.

**Figure 3:**
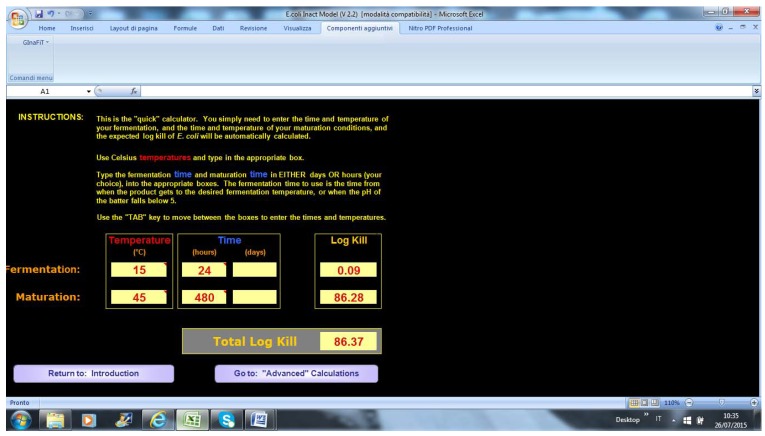
Output in MLA- quick calculation.

**Figure 4:**
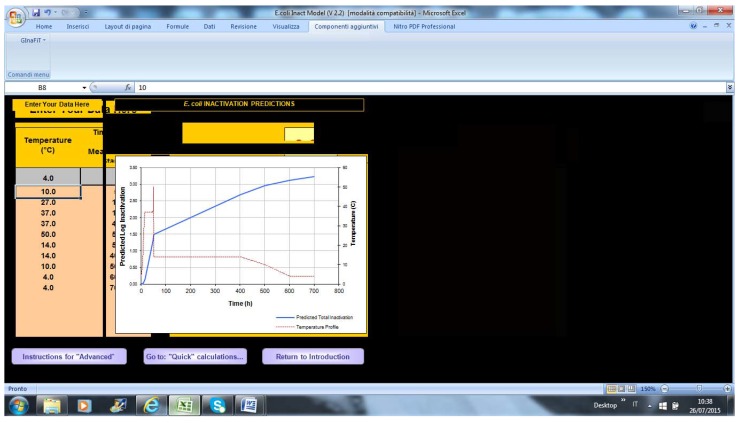
Advanced calculation in MLA.

The end-user is required to input the fermentation temperature and duration of maturation. The tool provides two outputs: log-inactivation of *E. coli* throughout fermentation, and maturation (Figure 3 and Figure 4).

Figure 4 shows the screen for advanced calculations; on the left column, the end-user can paste the data from a data logger and see the temperature profile and point-by-point inactivation of *E. coli* on the right.

### 3.3. AMIF Process Lethality Determination Spreadsheet

**Figure 5:**
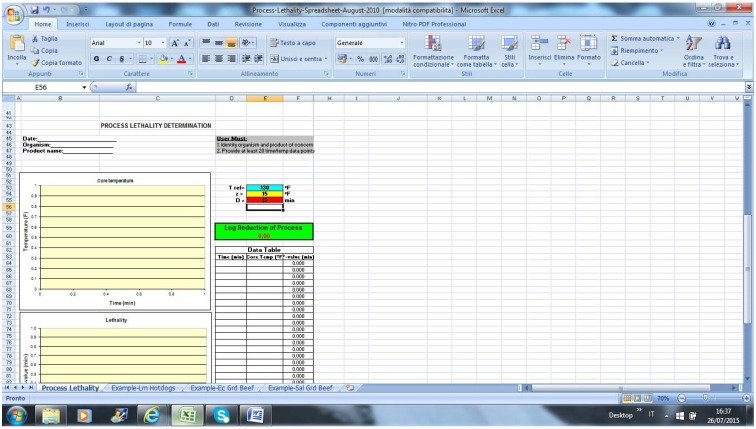
Screen of lethality spreadsheet (released August 2010).

This is an Excel tool that evaluates the lethality associated with a thermal process. The end-user should add the D and z value of the test microorganism, the reference temperature, and at least 20 data points from a data logger (temperature *vs*. time); the main outputs are the thermal profile and lethality picture, that is, log reduction as a function of time. Figure 5 shows the main screen of the tool.

### 3.4. DMFit

**Figure 6:**
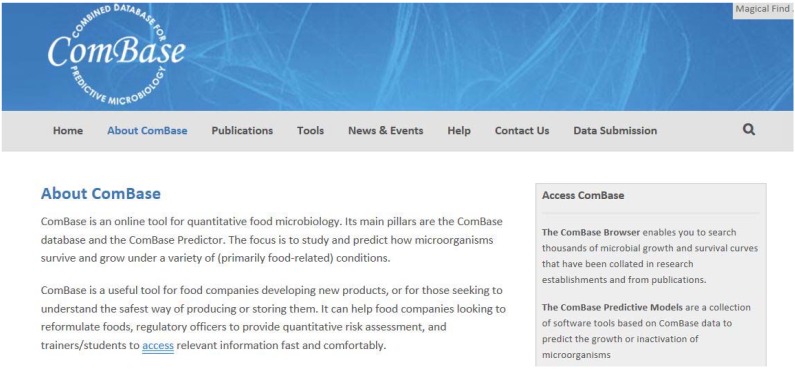
ComBase screen.

One of the most important and valuable tools in predictive microbiology is ComBase [29]; it includes a database with 50,000 records on how microorganisms behave in different environments (ComBase Browser) and some models to predict growth and inactivation (ComBase Predictor) (Figure 6).

**Figure 7:**
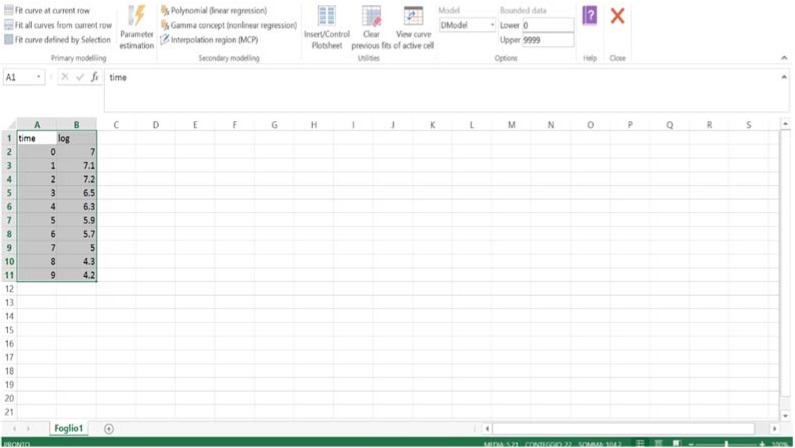
DMFit screen.

From the link “tools”, end-users can download some ComBase tools (Combase demo for Excel, DMFit, and Perfringens Predictor); moreover, links are provided to tools external to ComBase. DMFit for Excel is free software and works as an add-in-component. Once downloaded and installed, on the right top of the screen there is the link to the software (Figure 7).

The end user needs only to write cell count as a function of time and select and click on the option “fit curve defined by selection”. The software fits the data by using the model by Baranyi and Roberts [30].

**Figure 8:**
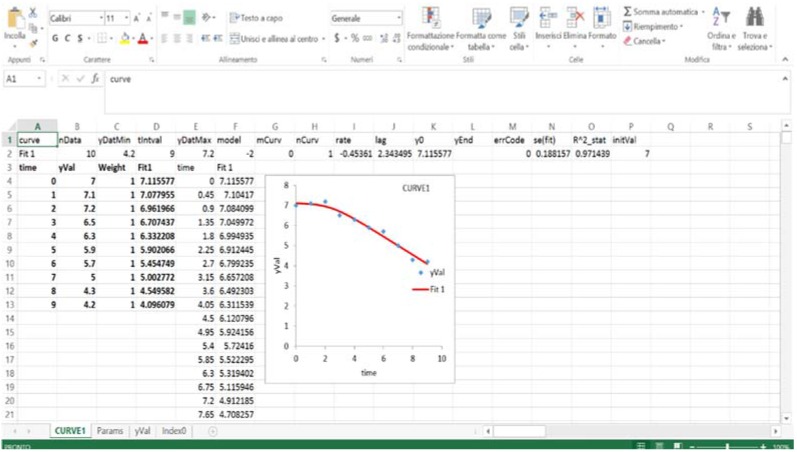
DMFit output.

The output is a new sheet containing the curve and the fitting parameters, along with some indices on the significance of the model. DMFit can also fit growth curves and build some simple secondary models (Figure 8).
